# The influence of prenatal exercise modes on resting maternal blood lactate

**DOI:** 10.14814/phy2.70444

**Published:** 2025-07-07

**Authors:** Alex Claiborne, Filip Jevtovic, Ericka M. Biagioni, Breanna Wisseman, Brittany Roenker, Kara Kern, Dylan Steen, Lindsey Rossa, Caitlyn Ollmann, Samantha Mcdonald, Cody Strom, Edward Newton, James Devente, Steven Mouro, David Collier, George A. Kelley, Jill Maples, Perrie O'Tierney‐Ginn, Nicholas T. Broskey, Joseph A. Houmard, Linda E. May

**Affiliations:** ^1^ Department of Kinesiology Nutrition and Health, Miami University Oxford Ohio USA; ^2^ Department of Kinesiology East Carolina University (ECU) Greenville North Carolina USA; ^3^ Human Performance Laboratory, ECU Greenville North Carolina USA; ^4^ East Carolina Diabetes & Obesity Institute, ECU Greenville North Carolina USA; ^5^ Department of Kinesiology University of Rhode Island Kingston Rhode Island USA; ^6^ Department of Applied Exercise Science Gannon University Erie Pennsylvania USA; ^7^ Department of Exercise and Sport Science University of Mount Olive Mount Olive North Carolina USA; ^8^ School of Kinesiology and Recreation Illinois State University Normal Illinois USA; ^9^ Department of Kinesiology and Sport University of Southern Indiana Evansville Indiana USA; ^10^ Department of Obstetrics & Gynecology ECU Greenville North Carolina USA; ^11^ Department of Pediatrics ECU Greenville North Carolina USA; ^12^ School of Public and Population Health and Department of Kinesiology Boise State University Boise Idaho USA; ^13^ Department of Obstetrics and Gynecology University of Tennessee Knoxville Tennessee USA; ^14^ Mother Infant Research Institute Tufts Medical Center Boston Massachusetts USA; ^15^ Department of Obstetrics & Gynecology Tufts University Boston Massachusetts USA

**Keywords:** exercise, lactate, obesity, overweight, pregnancy

## Abstract

Resting lactate concentration in venous blood is a commonly used indicator of metabolic disease risk. Regular exercise during pregnancy improves maternal metabolic health; however, it is unknown if maternal exercise regulates resting lactate concentration. We aimed to elucidate the effects of three different modalities of exercise during pregnancy on blood lactate in pregnant women. This is a secondary analysis of data from three blinded, prospective, randomized controlled trials. Pregnant women were randomized to control or exercise. Exercisers underwent ~24 weeks of supervised aerobic, resistance, or combination exercise between 12–16 and 37–40 weeks gestation. Fasted resting maternal blood lactate was collected at 16 and 36 weeks of gestation. Although lactate increased 0.5 mmol/L in controls across gestation, this rise was blunted in exercisers (*p* = 0.01). Pre‐pregnancy BMI was correlated with blood lactate in controls (*p* < 0.05, *R* ^2^ = 0.20) but not in exercisers (*p* > 0.05, *R* ^2^ = 0.01). Exercisers with overweight or obesity had lower 36‐week lactate (*p* = 0.001), particularly in aerobic and combination (*p* = 0.03; *p* = 0.006, respectively). These findings show that exercise helps control the BMI‐associated rise in maternal lactate seen in gestation and highlights the importance of exercise in women with overweight or obesity.

## 1 INTRODUCTION

Obesity during pregnancy increases the long‐term cardiometabolic health risk of mother and offspring (Langley‐Evans et al., 2022). Blood biomarkers such as glucose, insulin, and lactate have been shown to reflect cellular metabolism and insulin resistance; thus, as markers of metabolic health, these hold the potential to provide early risk management in pregnant women (Badon et al., 2018; Haddad et al., 2023; Lorenzo‐Almorós et al., 2019). An increase in venous blood lactate concentration reflects poor mitochondrial metabolism in skeletal muscle cells, thus acting as a quick and easy indicator of an individual's overall metabolic health. As the impairment in aerobic metabolism, namely oxidative phosphorylation, seen in states of reduced mitochondrial function underlies the development of metabolic disease, blood lactate is therefore commonly used to indicate metabolic disease risk (Broskey et al., 2024; Jones et al., 2019; Schapira, 2006). When collected at rest, lactate levels are used for prognosis across disease types, which commonly stem from overweight or obesity (Andersen et al., 2013), with higher lactate being associated with a worse disease state. In obesity and type 2 diabetes, for example, lactate levels are commonly >1.0 mmol/L (Chen et al., 1993), compared to ~0.8 mmol/L in non‐obese persons. However, in healthy pregnancy, lactate typically is <2 mmol/L outside of labor (Peguero et al., 2019).

Previous research has targeted the evaluation of maternal resting blood lactate for diabetes management during pregnancy, given its ease of measurement and implications for assessing underlying metabolism (Metz et al., 2005; Nagalakshmi, 2016). Given the predisposition to the development of metabolic disease, this measurement could be of particular importance in women with overweight or obesity. Acute exercise causes a transient increase in mitochondrial metabolism (Ejaz et al., 2023), and chronic exercise training is known to upregulate mitochondrial pathways (Egan & Zierath, 2013), thereby improving aerobic metabolism and reducing resting blood lactate (Broskey et al., 2024). In overweight individuals, previous research from our laboratory has shown up to a 16% reduction in resting lactate after chronic exercise training (Jones et al., 2019). Still, this effect is yet to be tested specifically in women, whether gravid or non‐gravid. It therefore remains unknown whether maternal exercise is an effective strategy for reducing resting blood lactate later in gestation (~36 weeks) especially in women with overweight or obesity. Furthermore, research regarding the utilization of different exercise types to reduce blood lactate in pregnancy is yet to be conducted.

Ultimately, it is important to determine whether prenatal exercise is effective in improving mitochondrial metabolism to decrease future maternal metabolic risk. To this end, we are interested in whether resting blood lactate, like other biomarkers of cardiometabolic health, is altered during pregnancy in women with and without overweight or obesity (OWOB). Therefore, this study aims to (1) determine the influence of prenatal exercise on maternal lactate levels in late pregnancy, (2) the influence of prenatal exercise on maternal lactate levels in late pregnancy in women with and without OWOB, and (3) determine the influence of aerobic, resistance, and combination exercise specifically. We hypothesized that the increase in blood lactate would be higher in late gestation in controls compared to exercisers, and this difference would be stronger in women with OWOB. Further, we expected this blunted rise in lactate to be present in all exercisers, regardless of type (aerobic, resistance, or combination).

## 2 MATERIALS AND METHODS

### 2.1 Study participants

This is a secondary analysis of maternal blood lactate from three prospective, randomized control trials (RCTs) investigating the influence of prenatal exercise types on offspring health outcomes. The current analysis will examine the influence of prenatal exercise (*n* = 140) versus control (*n* = 36) on maternal blood lactate in early and late pregnancy, as well as the effect of exercise group (AE aerobic *n* = 48, RE resistance *n* = 44, AERE combination *n* = 48). To assess these aims, pregnant women with HW (healthy‐weight) or OWOB (overweight or obesity) exercised with trained supervision for ~24 weeks of pregnancy, that is, from 12 to 16 weeks through 37 to 40 weeks, three times a week for 50 min per week at a moderate intensity. All protocols were approved by the East Carolina University (ECU) Institutional Review Board. Women enrolled in these studies met the following criteria: clearance from a health care provider to participate in physical activity; between 18 and 40 years of age; pre‐pregnancy body mass index (BMI; kg m^−2^) ≥18.5 kg m^−2^; singleton pregnancy; ≤16 weeks gestation; no current alcohol or tobacco use. Pre‐pregnancy BMI was a self‐report measure collected before enrollment. Criteria for exclusion included smoking, known pre‐existing conditions (i.e., diabetes mellitus, hypertension, cardiovascular disease, comorbidities, systemic lupus erythematosus), and/or medications known to affect fetal growth and well‐being.

### 2.2 Ethics statement

The three included studies used data from birth records collected from participants enrolled in an initial pilot RCT study, a second RCT (ClinicalTrials.gov Identifier: NCT03517293), and a third RCT (ClinicalTrials.gov Identifier: NCT03838146). Approval for these studies was obtained from the East Carolina University Institutional Review Board. Written informed consent was obtained from each participant upon enrollment. All experimental procedures were conducted at East Carolina University.

### 2.3 Pre‐intervention Exercise Testing and Randomization

After study enrollment, participants completed a submaximal treadmill test to determine aerobic capacity and calculate the target heart rate (THR) range for moderate‐intensity training. Peak oxygen consumption (VO_2_ peak) was estimated via the modified Balke protocol, a 2‐min stage progressive treadmill test performed to 85%–90% of an individual's estimated maximum heart rate, which has been previously validated for pregnant women (Mottola, 2008). To minimize exposure risk after the start of the COVID‐19 pandemic, women recruited between March 2020 and October 2021 had THR zones for aerobic exercise determined via a formula based on their pre‐pregnancy physical activity level and age (Mottola, 2008). THR zones for exercise components corresponded to maternal HR at 60 to 80% of maximal oxygen consumption, that is, moderate intensity. Next, participants were randomized via computerized sequencing (GraphPad, Boston, USA) to aerobic, resistance, combination (aerobic and resistance), or a stretching/breathing attention‐control group.

### 2.4 Exercise intervention

All participants were supervised by trained exercise instructors in ECU university facilities following a standard protocol. All sessions started at 16 weeks gestation, and women were scheduled three times weekly until delivery (Birsner & Gyamfi‐Bannerman, 2015). All participants' sessions included a 5‐min warm‐up, 50 min of their randomized group activity, and a 5‐min cool‐down. To ensure proper intensity was achieved during exercise sessions, the Borg scale rating of perceived exertion (RPE 6–20) and the “talk test,” that is, ensuring a participant was exercising just beyond a conversational pace, were used (Webster & Aznar‐Laín, 2008). HR monitoring (Polar FS2C, Kempele, Finland) ensured appropriate target HR ranges were maintained; target HR zones validated for pregnant women were utilized (Mottola, 2008). The intervention was progressive in that participant workload increased with fitness, as reflected by maintaining target HR.

The aerobic exercise (AE) group completed moderate‐intensity training on treadmills, ellipticals, recumbent bicycles, rowing, and/or stair‐stepping equipment. To maintain the appropriate HR zone, speed and grade were adjusted on the treadmill, and resistance and speed levels were adjusted on the elliptical and bicycle. The resistance exercise (RE) group completed sessions of two to three sets, aiming for 12 repetitions of each exercise at ~60% of 1 repetition maximum (1‐RM) (Moyer et al., 2016). Exercises for RE were performed in a circuit with minimal rest (5–10 s) using seated machines (Cybex) (i.e., leg extension, leg curl, shoulder press, chest press, triceps extension, latissimus dorsi pulldown), dumbbells (i.e., biceps curls, lateral shoulder raises, front shoulder raises), resistance bands, dumbbells, exercise balls, benches, and/or mats. The combination exercise (AERE) group performed half of the aerobic protocol and half of the resistance protocol exercises in five circuits, lasting 4.5–5 min each. Resistance exercises were performed aiming for 12 repetitions (same exercises and equipment as the RE group), while aerobic exercises were performed on the same equipment as the AE group.

### 2.5 Maternal covariates

Maternal age, gravida, parity, pre‐pregnancy weight, and height, as well as race and ethnicity were collected from pre‐screening eligibility questionnaires. Pre‐pregnancy BMI (healthy BMI 18.0–24.99; Overweight 24.5–29.99, Obese ≥30) was calculated using height (m), measured by stadiometer, and weight (kg) collected from the pre‐screening eligibility questionnaire. Maternal pre‐pregnancy BMI was calculated via the following established equation: [weight (kg)]/[height (m)]^2^.

### 2.6 Maternal blood lactate

At 16 and 36 weeks gestational age, women reported to our laboratory in the fasted state, having not performed exercise or eaten in the previous 8 h, and maternal blood was obtained via fingerstick and analyzed for lactate concentration (mmol/L) with a portable lactate analyzer (Nova Biomedical Lactate Plus, Waltham, MA, USA).

### 2.7 Statistical analysis

Resting blood lactate was analyzed at 16 and 36 weeks, and the change in blood lactate between 16 and 36 weeks gestation was analyzed as a separate variable. Pregnant women were grouped based on exercise group allocation (AE, RE, AERE, CON) and also based on pre‐pregnancy BMI status (HW ≤24.9 kg m^−2^, OWOB ≥25.0 kg m^−2^). Independent samples *t*‐tests were performed at each timepoint comparing exercise and control, then repeated in subjects qualifying as HW and OWOB BMI status. To test for the effect of exercise group (AE, RE, AERE) versus control, two‐way ANOVA compared blood lactate at both timepoints. To test for an effect of exercise on the relationship between maternal BMI and blood lactate, Pearson product–moment correlations were run between maternal pre‐pregnancy BMI and maternal lactate at 36 weeks for controls and exercisers. Significance for all tests was accepted at the *α* = 0.05 level. Correlations and *t*‐tests were performed using SPSS software (version 28.0.1.1, SPSS Inc. IBM Corp., Chicago, IL, USA) software.

## 3 RESULTS

Of 366 women who enrolled in the studies, 166 women had lactate measured at 16 weeks, 116 women at 36 weeks, and 104 had both time points for calculation of change scores (16–36 weeks). Women in this analysis were, on average, 29 years of age, of various BMIs (average: 26.3 kg/m^2^), and had full‐term pregnancies (average: 39.3 weeks; Table 1). Of the women included in the final analysis, 58% were classified as OWOB (Table S1), and 21% identified as Black or Indigenous People of Color (BIPOC).

**TABLE 1:** Maternal characteristics across exercise type.

Maternal characteristic	Control *n* = 36	Aerobic *n* = 48	Resistance *n* = 44	Combination *n* = 48	*p*
Age (years)	30.4 ± 4.4	30.2 ± 4.7	31.5 ± 3.8	30.4 ± 4.4	0.*49*
Gravida	2 (1,4)	2 (1,5)	2 (1,6)	2 (1,4)	0.*69*
Parity	1 (0,3)	1 (0,3)	1 (0,3)	0 (0,2)	0.*14*
% BIPOC	28	25	20	15	0.*47*
Pre‐pregnancy					
BMI kg m^−2^	26.9 ± 4.9	26.1 ± 4.8	24.6 ± 3.7	26.9 ± 4.9	0.*08*
% OWOB	58	53	40	58	0.*26*
36 weeks gestation					
BMI kg m^−2^	26.0 ± 3.5	26.6 ± 4.9	25.6 ± 3.8	26.8 ± 4.0	0.*71*
% OWOB	60	61	50	63	0.*76*

*Note*: Maternal characteristics measured before commencement of exercise (12–16 weeks of gestation). BIPOC Black or Indigenous People of Color. Data are mean ± SD.

Abbreviations: BMI, Body Mass Index; OWOB, with Overweight or Obesity.

### 3.1 Exercise influence on maternal lactate, all BMIs

**FIGURE 1:**
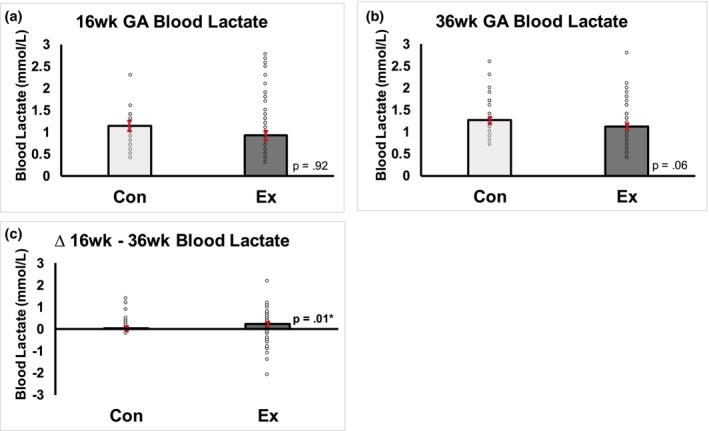
Prenatal exercise prevents increase in blood lactate during gestation. Exercising women saw no difference in resting blood lactate at 16 weeks gestation when compared to control (a), yet a trend for significant difference between the groups was observed at 36 weeks gestation (b), thus suggesting that exercise prevented the increase in blood lactate during gestation (c).

**FIGURE 2:**
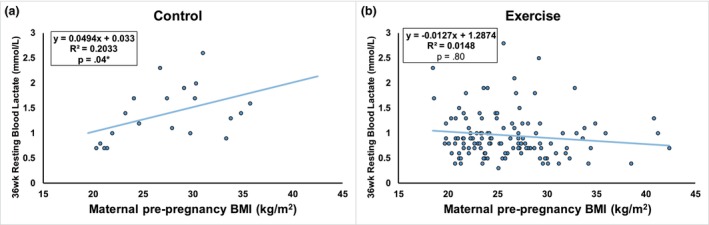
Prenatal exercise prevents BMI‐associated increase in blood lactate. In control subjects, individuals with higher pre‐pregnancy BMI saw relatively higher blood lactate levels at 36 weeks gestational age (a; *R* ^2^ = 0.20, *p* = 0.04). Prenatal exercise completely prevented this association between pre‐pregnancy BMI and blood lactate in pregnant women (b; *R* ^2^ = 0.01, *p* = 0.80).

**FIGURE 3:**
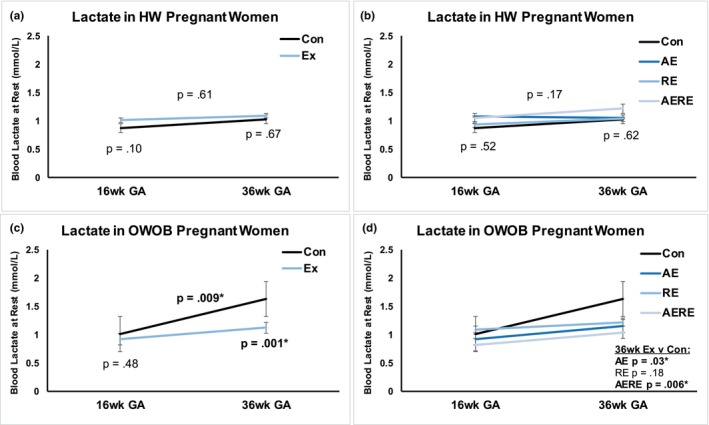
Exercise prevents gestational rise in blood lactate in OWOB. In healthy weight (HW) women, no differences in blood lactate were observed between control and exercise (a) or any of the exercise groups (b). However, in women with overweight or obesity (OWOB), exercisers saw significantly lower 36‐week lactate (c), in all exercise groups except resistance (d). This suggests that in OWOB particularly, aerobic (AE) or combination (AERE) exercise effectively prevents the gestational rise in resting blood lactate.

There was no difference in resting blood lactate at 16 weeks gestation in exercisers compared to controls (Figure 1a, *p* = 0.92) before the intervention; a trend for a significant difference between EX and CON was observed at 36 weeks gestation (Figure 1b, *p* = 0.06), which resulted in a greater increase in blood lactate from 16 to 36 weeks of gestation in controls compared to exercising mothers. In CON, there was a positive association between pre‐pregnancy BMI and maternal lactate levels at 36 weeks gestational age (Figure 2a, *R* ^2^ = 0.20, *p* = 0.04); however, this was not observed in EX (Figure 2b, *R* ^2^ = 0.01, *p* = 0.80). In HW women, no differences in blood lactate EX vs. CON were observed (Figure 3a, 16 weeks *p* = 0.10; 36 weeks *p* = 0.87). No influence of exercise type was seen when considering all BMIs (Figure 3b).

### 3.2 Exercise influence on maternal lactate, OWOB only

**FIGURE 4:**
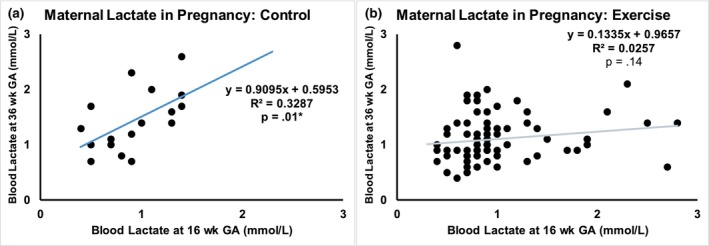
Maternal lactate through gestation. In control women, blood lactate was correlated between 16 and 36 weeks GA (a), while exercise prevented this association (b).

In women with overweight or obesity (OWOB), 36‐week lactate was significantly lower in EX versus CON (Figure 3c, *p* = 0.001). In CON, blood lactate was positively correlated between 16 and 36 weeks gestational age (GA) (Figure 4a; *R* ^2^ = 0.33, *p* = 0.01); women with higher blood lactate at 16 weeks GA also had higher lactate at 36 weeks GA. However, in exercising women, there was no correlation between lactate at 16 and 36 weeks GA, suggesting exercise might attenuate the gestational rise in lactate (Figure 4b; *R* ^2^ = 0.03, *p* = 0.14).

### 3.3 Exercise type influence on maternal lactate, OWOB only

In women with OWOB, there was a significant difference in 36‐week lactate among AE and AERE exercise groups (Figure 3d, RE *p* = 0.18; AE *p* = 0.03; AERE *p* = 0.006). This suggests that in OWOB, particularly, aerobic (AE) or combination (AERE) exercise effectively prevents the gestational rise in resting blood lactate.

## 4 DISCUSSION

The current study aimed to test for the effects of exercise during pregnancy on fasted maternal blood lactate levels in the first and third trimesters of pregnancy. We hypothesized that blood lactate would increase higher during gestation in women with overweight and obesity (OWOB) when compared to healthy weight women (HW). We also expected exercise of any type would be associated with a reduction in the increase in resting blood lactate seen during pregnancy. More specifically, we hypothesized this relationship to be apparent in HW and more strongly in women with OWOB. We confirmed firstly that pre‐pregnancy BMI was positively associated with the rise in blood lactate seen at 36 weeks gestational age, such that women with OWOB saw a significant increase in resting lactate, while HW did not. Prenatal exercise was shown to prevent this rise in lactate in OWOB subjects in all exercise types included (aerobic AE, combination AERE) except for resistance only (RE). Thus, we believe exercise during pregnancy to be a strong tool to manage fasting lactate in women with OWOB.

Among this population of pregnant women, we observed a significant positive relationship between pre‐pregnancy BMI and resting blood lactate at 36 weeks gestation. Thus, women with OWOB showed a higher blood lactate measure, which has been seen previously as a marker for altered substrate utilization and insulin resistance (Metz et al., 2005; Nagalakshmi, 2016). This indicates that the direct relationship between BMI and a marker of metabolic health risk exists in pregnant women, and highlights an important area of focus for future research. This finding could have implications for postpartum health in mothers, as well as the risk of complications in offspring, as this increased blood lactate has also been found in afflicted fetuses, who are also born with lower umbilical vein oxygen saturation (Taricco et al., 2009).

Exercise prevented the rise in resting blood lactate in pregnant women typically seen between 16 and 36 weeks GA. Furthermore, in exercisers alone, the influence of maternal BMI on resting blood lactate disappeared, while higher BMI was associated with higher lactate in controls at 36 weeks GA. This finding indicates that ~6 months of structured exercise can improve metabolic health in pregnant women. Of note, we found this exercise effect to be stronger in women reporting OWOB pre‐pregnancy, as these women also saw significantly increased resting lactate compared to HW at 36 weeks gestation. Still, in pregnant women with OWOB who exercised for ~24 weeks of pregnancy, meeting recommended levels of 150 min per week at moderate intensity, exercise prevented the rise in resting lactate at 36 weeks gestation; thus supporting that prenatal exercise should continue to be recommended as a powerful strategy to reduce metabolic risk in pregnancy (Davenport et al., 2018; Jevtovic, Zheng, et al., 2023; McDonald et al., 2021; Mottola, 2008).

While benefits to maternal metabolic health were seen among most exercisers, a significant effect of exercise was limited to improvement of blood lactate in OWOB aerobic (AE) and combination (AERE) exercisers, and not resistance only (RE). This finding was surprising, as we expected all types to incur a significant benefit, but based on the stimulation of oxidative phosphorylation prioritized in aerobic and combination exercise modalities, this finding can be partially explained (Jones et al., 2019). Furthermore, we highlight that even without a focus on aerobic exercise, resistance exercise did lead to a non‐significant reduction in lactate at 36 weeks GA.

### 4.1 Strengths & Limitations

Our findings are strengthened by the supervised randomized controlled exercise intervention study with trained professionals. To the best of our knowledge, this is the first report of exercise during pregnancy preventing a gestational rise in resting blood lactate across gestation and, therefore, has implications for future studies in pregnant populations. Furthermore, consideration of these findings in the scope of a diverse study sample (22% BIPOC), as families that identify as BIPOC have shown impaired glucose metabolism and greater metabolic health risk (Bower et al., 2019; Jevtovic, Lopez, et al., 2023). In addition to strengths, there are several potential limitations. First, prenatal exercise was controlled as part of a prescribed exercise dose; therefore, limiting our ability to study a dose–response effect, but we believe future analysis should account for this. Second, our sample size was limited relative to some larger epidemiological studies. Third, this was a secondary analysis of data rather than a focused study recruiting women with increased resting blood lactate. Further, our women did not develop GDM, so our findings should be taken in the context of women relatively closer to developing this metabolic complication. Finally, the influence of other stressors during pregnancy, for example, altered sleep and nutritional habits, physical discomforts, and stress and anxiety, were not tested in this study and should be assessed in future work. Therefore, we recommend larger intervention studies are needed, specifically recruiting women at strong risk of developing GDM by 28 weeks gestational age to confirm our findings. Our results will better inform sample size calculations for these larger intervention studies.

## 5 CONCLUSION

Past research has shown a strong relationship between maternal resting blood lactate, offering an easy‐to‐measure predictive clinical tool to be studied. Our findings show that supervised exercise training decreases the BMI‐associated rise in blood lactate in pregnant women, and therefore, data supports that exercise, especially incorporating an aerobic component, is an effective strategy to mitigate metabolic risk in these women. These findings highlight the importance of obstetricians encouraging women at risk of developing metabolic disorders to exercise during pregnancy at recommended levels. Future research should focus on the mechanisms underlying this association in vascular and skeletal muscle tissue, and whether prenatal exercise influences postpartum metabolic health.

## AUTHOR CONTRIBUTIONS

**Alex Claiborne**: visualization, writing—original draft, writing—review and editing, formal analysis, investigation. **Filip Jevtovic**: data curation, software, writing—review and editing. **Ericka M. Biagioni**: writing, review and editing. **Breanna Wisseman**: data curation, software, writing—review and editing. **Brittany Roenker**: writing—review and editing. **Kara Kern**: data curation, software, writing—review and editing. **Dylan Steen**: data curation, software, writing—review and editing. **Lindsey Rossa**: project administration, writing—review and editing. **Caitlyn Ollmann**: project administration, writing—review and editing. **Samantha McDonald**: data curation, software, writing—review and editing. **Cody Strom**: data curation, software, writing—review and editing. **Edward Newton**: project administration, writing—review and editing. **Christy Isler**: project administration, writing—review and editing; **James deVente**: project administration, writing— review and editing. **Steven Mouro**: project administration, writing—review and editing. **David Collier**: project administration, writing—review and editing. **Devon Kuehn**: project administration, writing—review and editing. **George A. Kelley**: project administration, writing—review and editing. **Jill Maples**: writing ‐ review and editing. **Perrie O'Tierney** **‐Ginn**: writing ‐ review and editing. **Nicholas T. Broskey**: investigation, writing—review and editing. **Joseph A. Houmard**: investigation, writing—review and editing. **Linda E. May**: conceptualization, data curation, funding acquisition, investigation, methodology, project administration, resources, software, supervision, validation, writing—review and editing.

## FUNDING INFORMATION

This research was supported by American Heart Association Grant #15GRNT24470029 (PI Linda May) and an internal grant from East Carolina University.

## CONFLICT OF INTEREST STATEMENT

The authors report no conflict of interest.

## ETHICS STATEMENT

This study used data from participants enrolled in a randomized controlled trial (ClinicalTrials.gov Identifier: NCT03517293). Approval for this study was obtained from the East Carolina University Institutional Review Board. All experimental procedures were conducted at East Carolina University.

## Supporting information


**Table S1.** Maternal characteristics by intervention group and BMI classification.

## Data Availability

Data will be made available upon request.
