# Supplementary material for: The influence of prenatal exercise modes on resting maternal blood lactate
Source: Physiol Rep. 2025 Jul 7;13(13):e70444. doi: 10.14814/phy2.70444 (PMC12234768; doi:10.14814/phy2.70444)
Supplement: Supplementary file 1 — Table S1. Maternal characteristics by intervention group and BMI classification. [file PHY2-13-e70444-s001.docx]

**Supplemental Table S1** Maternal Characteristics by Intervention Group and BMI Classification.

| **HW Only** | **CON**  *n=36* | **EX**  *n=115* | **AE**  *n=48* | **RE**  *n=44* | **AERE** *n=48* |
| --- | --- | --- | --- | --- | --- |
| Maternal Age *yrs* | 29.7 ± 4.1 | 30.3 ± 4.2 | 29.8 ± 4.3 | 31.2 ± 4.3 | 30.3 ± 4.1 |
| Pre-Pregnancy BMI *kg/m^2^* | 21.9 ± 1.5 | 22.3 ± 1.6 | 22.2 ± 1.7 | 22.2 ± 1.5 | 22.5 ± 1.6 |
| Gravida | 1 (1,4) | 1 (1,6) | 1 (1,4) | 1 (1,6) | 1 (1,4) |
| Parity | 0 (0,3) | 0 (0,3) | 0 (0,2) | 0 (0,3) | 0 (0,2) |
| GWG *kg* | 9.3 ± 3.0 | 10.5 ± 3.4 | 10.0 ± 2.8 | 10.3 ± 4.2 | 11.3 ± 3.5 |
| GFG *kg* | 0.99 ± 2.1 | 0.95 ± 2.0 | 0.40 ± 2.1 | 0.71 ± 1.8 | 2.07 ± 1.8 |
| **OWOB Only** | **CON**  *n=34* | **EX**  *n=53* | **AE**  *n=21* | **RE**  *n=13* | **AERE** *n=19* |
| Maternal Age *yrs* | 28.9 ± 5.0 | **30.4 ± 4.2*** | 30.3 ± 4.4 | 31.2 ± 3.8 | 30.0 ± 4.4 |
| Pre-Pregnancy BMI *kg/m^2^* | 30.8 ± 4.5 | 29.9 ± 4.8 | 29.8 ± 5.0 | 30.1 ± 5.9 | 30.0 ± 3.9 |
| Gravida | 2 (1,6) | 1 (1,5) | 1 (1,5) | 2 (1,5) | 1 (1,4) |
| Parity | 1 (0,4) | 0 (0,3) | 0 (0,3) | 1 (0,3) | 0 (0,2) |
| GWG *kg* | 9.8 ± 4.7 | 10.8 ± 4.2 | 11.1 ± 4.5 | 11.3 ± 4.1 | 9.9 ± 4.0 |
| GFG *kg* | -0.08 ± 2.7 | 0.99 ± 2.4 | 0.06 ± 2.3 | **2.66 ± 1.7*** | 0.25 ± 2.5 |
| Data reported as mean ± SD. Maternal characteristics measured before commencement of exercise (12 – 16 weeks of gestation) or documented at birth (GWG, GFG). BMI body mass index in kg/m^2^ (HW < 25.0, OWOB > 25.0); GWG gestational weight gain; GFG gestational fat gain. CON Attention Control, AE Aerobic Exercise, RE Resistance Exercise, AERE Combination Exercise. Tukey post-hoc results: *p < .05 vs. control. | | | | | |
